# 1056. Correlates of Omicron SARS-CoV-2 viral load: diagnostic and clinical implications

**DOI:** 10.1093/ofid/ofac492.897

**Published:** 2022-12-15

**Authors:** Simon Pollett, Stephanie A Richard, Anthony C Fries, Allison M Malloy, Anuradha Ganesan, Jeffrey Livezey, David Saunders, Nikhil Huprikar, Rupal Mody, Katrin Mende, David A Lindholm, Catherine M Berjohn, Julia S Rozman, Milissa Jones, Christopher Colombo, Rhonda E Colombo, David Tribble, Mark P Simons, Brian Agan, Timothy Burgess

**Affiliations:** Infectious Disease Clinical Research Program, Department of Preventive Medicine and Biostatistics, Uniformed Services University of the Health Sciences, Bethesda, MD, USA, Bethesda, Maryland; Infectious Disease Clinical Research Program, Department of Preventive Medicine and Biostatistics, Uniformed Services University of the Health Sciences, Bethesda, MD, USA, Bethesda, Maryland; U.S. Air Force School of Aerospace Medicine, Dayton, Ohio; Department of Pediatrics, Uniformed Services University of the Health Sciences, Bethesda, MD, USA, Bethesda, Maryland; Infectious Disease Clinical Research Program, Department of Preventive Medicine and Biostatistics, Uniformed Services University of the Health Sciences, Bethesda, MD, USA, Walter Reed National Military Medical Center, Bethesda, Maryland; Uniformed Services University of the Health Sciences, Bethesda, MD, USA, Bethesda, Maryland; Uniformed Services University of the Health Sciences, Bethesda, MD, USA, Bethesda, Maryland; Walter Reed National Military Medical Center, Bethesda, Maryland; William Beaumont Army Medical Center, El Paso, Texas; Infectious Disease Clinical Research Program, Department of Preventive Medicine and Biostatistics, Uniformed Services University of the Health Sciences, Bethesda, MD, USA, Bethesda, Maryland; Brooke Army Medical Center, Bethesda, Maryland; Naval Medical Center San Diego Division of Infectious Diseases, Infectious Disease Clinical Research Program, San Diego, CA; Infectious Disease Clinical Research Program, Department of Preventive Medicine and Biostatistics, Uniformed Services University of the Health Sciences, Bethesda, MD, USA, Bethesda, Maryland; Tripler Army Medical Center, Honolulu, Hawaii; Madigan Army Medical Center, Tacoma, Washington; Infectious Disease Clinical Research Program, Department of Preventive Medicine and Biostatistics, Uniformed Services University of the Health Sciences, Bethesda, MD, USA, The Henry M. Jackson Foundation for the Advancement of Military Medicine, Madigan Army Medical Center Division of Infectious Diseases, Tacoma, Washington; Infectious Disease Clinical Research Program, Department of Preventive Medicine and Biostatistics, Uniformed Services University of the Health Sciences, Bethesda, MD, USA, Bethesda, Maryland; Infectious Disease Clinical Research Program, Department of Preventive Medicine and Biostatistics, Uniformed Services University of the Health Sciences, Bethesda, MD, USA, Bethesda, Maryland; Infectious Disease Clinical Research Program, Department of Preventive Medicine and Biostatistics, Uniformed Services University of the Health Sciences, Bethesda, MD, USA, Bethesda, Maryland; Infectious Disease Clinical Research Program, Department of Preventive Medicine and Biostatistics, Uniformed Services University of the Health Sciences, Bethesda, MD, USA, Bethesda, Maryland

## Abstract

**Background:**

Omicron SARS-CoV-2 infections are associated with less frequent olfactory sensory loss and a predominance of pharyngitis symptoms compared to prior variants, with proposed diagnostic implications. We examined whether such symptomology predicts a higher RNA abundance in the oropharynx. We further investigated how age, symptom-day, vaccination history and clinical severity correlate with viral load to inform clinical prognostication and transmission modeling.

**Methods:**

The EPICC study is a longitudinal cohort of COVID-19 cases enrolled through U.S military medical treatment facilities. Demographic and clinical characteristics were measured with interviews and surveys. Nasopharyngeal (NP), oropharyngeal (OP) and nasal swabs (NS) were collected for SARS-CoV-2 qPCR and sequence genotyping. Multivariable linear regression models were fit to estimate the effect of anatomical site on SARS-CoV-2 RNA abundance (a proxy for viral load), adjusting for sampling time, vaccine history and host age.

**Results:**

We analyzed 77 sequence-confirmed Omicron cases; no BA.2 cases were detected. The median age was 38.8 years. 81.8% were vaccinated and 15.6% cases were hospitalized. 80.0%, 21.8%, and 65.5% reported nasal congestion, loss of smell or taste, and sore throat, respectively. The median RNA abundance was lowest in OP swabs (p < 0.001) (Fig 1). Linear regression confirmed that OP sampling was associated with lower viral load (p < 0.001). We further noted that greater age and symptom-day were independent correlates of viral load (Table 1). By bivariate analysis there was a trend toward lower RNA abundance in vaccinated subjects (p = 0.35). RNA abundance (at any site) was substantially higher in hospitalized (10634 N2 genome equivalents [GE]/reaction) versus outpatient cases (1419 N1 GE/reaction) but this was not statistically significant (p = 0.26).

**Fig 1.**
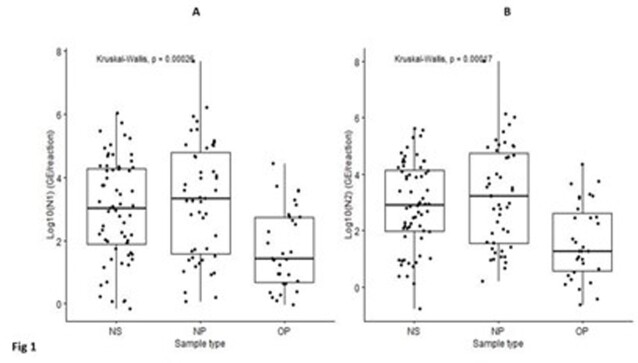
RNA abundance by upper respiratory swab anatomical location of collection (n = 142 swabs from n = 77 subjects)

**Figure:**
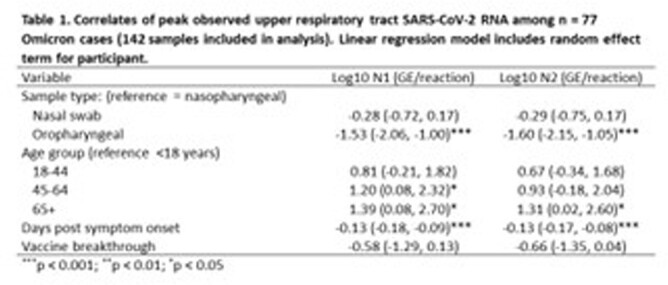

**Conclusion:**

We noted prevalent sore throat symptoms and infrequent sensory loss in Omicron cases. Despite this, viral load was highest in NP/NS collected swabs as has been noted in prior variants. We note an age correlation with RNA abundance, and provide a viral load decay rate which may be useful for transmission modeling. Vaccination and clinical severity may also correlate with Omicron viral load, as noted with prior SARS-CoV-2 variants.

**Disclosures:**

**Simon Pollett, MBBS**, Astra Zeneca: The HJF, in support of the USU IDCRP, was funded to conduct or augment unrelated Phase III Mab and vaccine trials as part of US Govt. COVID19 response **Julia S. Rozman, n/a**, Astra Zeneca: The HJF, in support of the USU IDCRP, was funded to conduct or augment unrelated Phase III Mab and vaccine trials as part of US Govt. COVID19 response **David Tribble, MD, DrPH**, Astra Zeneca: The HJF, in support of the USU IDCRP, was funded to conduct or augment unrelated Phase III Mab and vaccine trials as part of US Govt. COVID19 response **Mark P. Simons, PhD**, AstraZeneca: The HJF, in support of the USU IDCRP, was funded to conduct or augment unrelated Phase III Mab and vaccine trials as part of US Govt. COVID19 response **Timothy Burgess, MD, MPH**, AstraZeneca: The HJF, in support of the USU IDCRP, was funded to conduct or augment unrelated Phase III Mab and vaccine trials as part of US Govt. COVID19 response.

